# Sn-doped Bi_1.1_Sb_0.9_Te_2_S bulk crystal topological insulator with excellent properties

**DOI:** 10.1038/ncomms11456

**Published:** 2016-04-27

**Authors:** S. K. Kushwaha, I. Pletikosić, T. Liang, A. Gyenis, S. H. Lapidus, Yao Tian, He Zhao, K. S. Burch, Jingjing Lin, Wudi Wang, Huiwen Ji, A. V. Fedorov, Ali Yazdani, N. P. Ong, T. Valla, R. J. Cava

**Affiliations:** 1 Frick Chemistry Laboratory, Department of Chemistry, Princeton University, Princeton, New Jersey 08544, USA; 2 Department of Physics, Princeton University, Princeton, New Jersey 08544, USA; 3 Brookhaven National Laboratory, Condensed Matter Physics and Materials Science Department, Upton, New York 11973, USA; 4 X-ray Science Division, Advanced Photon Source, Argonne National Laboratory, Argonne, Illinois 60439, USA; 5 Department of Physics, University of Toronto, Toronto, Ontario, Canada M5S 1A7; 6 Department of Physics, Boston College, Boston, Massachusetts 02467-3804, USA; 7 Advanced Light Source, Lawrence Berkeley National Laboratory, Berkeley, California 94720, USA

## Abstract

A long-standing issue in topological insulator research has been to find a bulk single crystal material that provides a high-quality platform for characterizing topological surface states without interference from bulk electronic states. This material would ideally be a bulk insulator, have a surface state Dirac point energy well isolated from the bulk valence and conduction bands, display quantum oscillations from the surface state electrons and be growable as large, high-quality bulk single crystals. Here we show that this material obstacle is overcome by bulk crystals of lightly Sn-doped Bi_1.1_Sb_0.9_Te_2_S grown by the vertical Bridgman method. We characterize Sn-BSTS via angle-resolved photoemission spectroscopy, scanning tunnelling microscopy, transport studies, X-ray diffraction and Raman scattering. We present this material as a high-quality topological insulator that can be reliably grown as bulk single crystals and thus studied by many researchers interested in topological surface states.

## Abstract (web summary)

An ideal topological insulator possesses an insulating bulk and a unique conducting surface however such behaviour is typically inhibited by bulk conduction due to defects. Here, the authors show that Sn-doped Bi_1.1_Sb_0.9_Te_2_S grown by the vertical Bridgman technique might overcome this hurdle.

## Graphical abstract

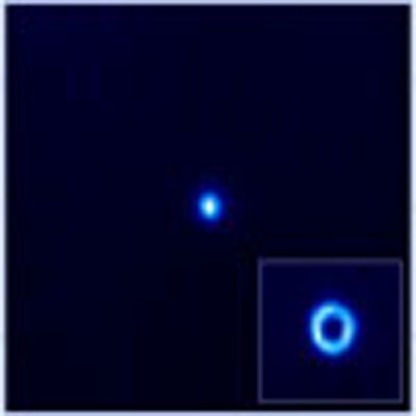

Topological insulators (TIs) display a new state of quantum matter^1^,^2^,^3^,^4^,^5^. In these three-dimensional, small band gap bulk insulators, metallic two-dimensional spin-momentum locked Dirac fermions exist on the surfaces. These fermions, exhibiting a quasi-linear energy versus wavevector dispersion relation near their Dirac point (DP), owe their existence and protection to the time-reversal symmetry^6^. Three-dimensional TIs have sparked wide interest in the research community due to both their fundamental scientific interest and the opportunities they offer for observing new electronic phenomena and properties that may be exploited for spintronics or other advanced electronic applications^7^,^8^,^9^,^10^,^11^,^12^,^13^. Unfortunately, however, the transport properties of the actual materials involved are typically dominated by the bulk electrons^5^,^14^,^15^,^16^, making the true electronic properties of the surface states (SSs) difficult to observe and exploit.

Bi_2_Se_3_ and Bi_2_Te_3_, with bulk band gaps of ∼300 and ∼150 meV, respectively, are members of the larger *M*_2_*X*_3_ (*M*=Sb, Bi; *X*=S, Se, Te) tetradymite family and are widely considered to be fundamental materials platforms for the investigation of topological SSs (TSSs). The best bulk Bi_2_Se_3_ crystals are heavily doped^16^, with their Fermi level in the bulk conduction band (BCB), and thus the SS cannot be characterized independently of interference from the bulk states and in Bi_2_Te_3_ the SS DP energy is below that of the top of the bulk valence band (BVB) and the crystals have large bulk carrier concentrations due to antisite defects^17^,^18^. Thus, neither of the simple materials is suitable for experimentally probing the intrinsic character of the TSS.

Efforts have been underway since the inception of the field, to find a material that is the best bulk single crystal TI for studying the intrinsic properties of the surface Dirac electrons^19^,^20^,^21^,^22^,^23^,^24^,^25^,^26^,^27^,^28^,^29^,^30^. So far, the primary desirable characteristics for the ideal bulk crystal material—to have a SS DP well isolated in energy from the bulk states and a very low bulk carrier concentration, both of which would allow for the study of the Dirac electrons at very low energies without interference from the bulk states, quantum oscillations from the TSS electrons and reliable growability as high-quality single crystals—have proven to be difficult, to satisfy in a single real bulk material. Until the current work, Bi_2_Te_2_Se, with well-controlled stoichiometry and defect chemistry, has been considered as the best bulk crystal material from this perspective, but its DP energy is below the maximum energy of the BVB by ∼60 meV, making it impossible to study the SS electrons close to the DP without interference from bulk states^20^,^24^,^25^,^26^,^27^. An alternative material is the quaternary solid solution (Bi_1−*x*_Sb_*x*_)_2_(Se_1−*y*_Te_*y*_)_3_ (refs 28, 29, 30), but the stoichiometry of that material is not fixed by any intrinsic thermodynamic material characteristic such as chemistry or structure, making it unsuitable as a general materials platform for the study of TSS. Although very important measurements have been performed on carefully prepared thin films of materials in the (Bi_1−*x*_Sb_*x*_)_2_(Se_1−*y*_Te_*y*_)_3_ family, the lack of a bulk material with excellent properties that can be reproducibly grown as large bulk single crystals has significantly limited the number of experimental research groups that can contribute to the development of the field.

Here we describe the design, growth and characterization of a material that we argue satisfies all the synthesis and electronic structure criteria to be an ideal bulk TI. This material is derived by combining concepts in crystal chemistry, defect chemistry, strain-mediated chemical stability, the effect of atomic electronegativities on the absolute energies of bulk electronic states and resonant level doping. The result is the stable, bulk-insulating, high-crystallinity single crystal material Bi_1.1_Sb_0.9_Te_2_S, grown by the vertical Bridgman technique (VBT) and doped with a very small percentage of Sn. Large crystals with excellent properties can be reproducibly grown. This material displays an SS DP energy fully isolated from the BVB and BCB, very-low bulk carrier concentrations, strong domination of the conductance by surface currents below 100 K and reproducibly observed quantum oscillations from the TSSs. Its growth, described here, will make the characterization of TSSs available to a wide community of researchers.

## Results

### Material design

**Figure 1:**
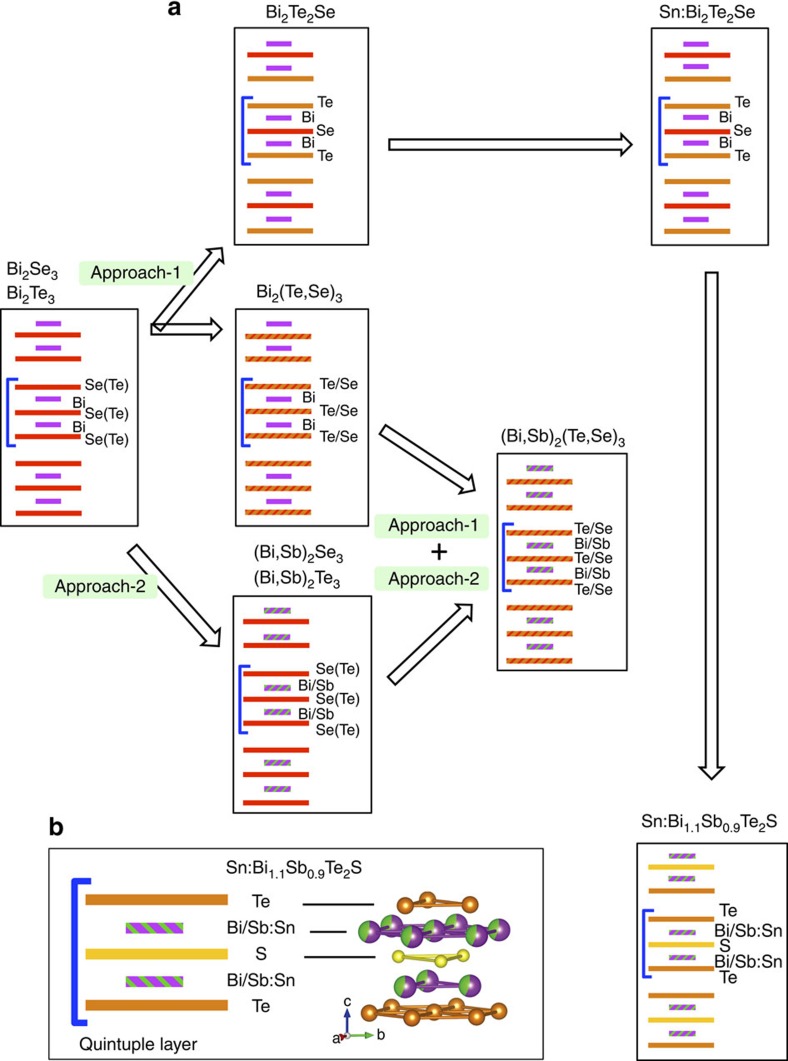
Evolution of tetradymite type TIs. (**a**) Schematic representation of the evolution of Bi_2_Se_3_, (*M*_2_*X*_3_) type TIs, through the two approaches (Approach-1 and Approach-2). The arrows represent approaches and progresses made in achieving better materials. The solid lines represent the single atomic layers and zebra lines represent multi-element layers. The purple, green, red, dark orange and yellow colours are taken, respectively, for Bi, Sb, Se, Te and S atoms. (**b**) The schematic of a QL for Sn:Bi_1.1_Sb_0.9_Te_2_S and a QL obtained by structure refinement of a powdered specimen. The colour fraction for Bi/Sb atoms is according to the composition obtained by unit cell refinement.

Figure 1a shows a schematic view of the materials design process that leads us to Bi_1.08_Sn_0.02_Sb_0.9_Te_2_S (Sn-BSTS). We start with Bi_2_Se_3_ and Bi_2_Te_3_, which are the simplest, although far from ideal, tetradymite-type TI materials. To obtain bulk insulating TI crystals based on these tetradymites, two main approaches and their combination have been employed. In Approach-1, starting from Bi_2_Se_3_, Te has been partially substituted for Se to form Bi_2_Te_2_Se (BTS), where the electron-donating Se vacancies in Bi_2_Se_3_ are controlled by confining the Se to the middle layer in the quintuple-layer (QL) structure or, alternatively, by the much less well-controlled random Bi_2_(Se_1−*x*_Te_*x*_)_3_ solid solution. In Approach-2, the composition of Bi-site is adjusted by Sb-Bi partial substitution. This method has the advantage of yielding materials, whose DP is isolated from the bulk states, but the defect chemistry is again difficult to control. By using both approaches simultaneously, yielding tetradymites of the type (Bi_1−*x*_Sb_*x*_)_2_(Se_1−*y*_Te_*y*_)_3_, the energy of the SS DP can be placed in the bulk band gap, and films and crystals can be grown with low bulk carrier concentrations at low temperature^28^,^29^,^30^. The disadvantage of this material is the difficulty in controlling its composition and therefore its defect chemistry, to grow large high-quality bulk single crystals.

Here we develop an alternative material system based on incorporating S rather than Se in the middle layer of Bi_2_Te_2_Se. The incorporation of S in place of Se in the middle layer of the tetradymite QL sandwich decreases the absolute energy of the valence band due to the higher electronegativity^31^ of S and makes the surface DP isolated in energy from the bulk states. This is a parallel materials development path to Approach-1. However, the formation of a thermodynamically stable compound of composition Bi_2_Te_2_S is not possible, due to crystallographic strain from layer size mismatch, as was early described by Pauling^32^ and so a decrease of the size of the Bi layers must be made through Approach-2 to obtain a stable material. Through experimentation we have found that the composition of that stable material is Bi_1.1_Sb_0.9_Te_2_S. This composition is a result of chemical synergism: the incorporation of S in the middle of the QL requires a contraction of the metal layers (the ones containing Bi and Sb, Sb is smaller than Bi) for size matching and selects a Bi/Sb ratio near 1:1 for stability (Fig. 1b). This is a very strong crystal-chemical driving force for stability and results in the optimal growth of this composition as large single crystals directly from a melt of the same composition. This material already has a low carrier concentration, but as a final step we employ the concept of resonant level doping, developed in previous work to optimize the properties of tetradymite thermoelectrics, where small amounts of Sn are known to act in that capacity^24^,^25^,^33^, to compensate for the last of the native defects present. We thus obtain a bulk-insulating ideal TI through 1% Sn substitution for Bi and Sb and develop the material Bi_1.08_Sn_0_._02_Sb_0.9_Te_2_S.

### Crystals

**Figure 2:**
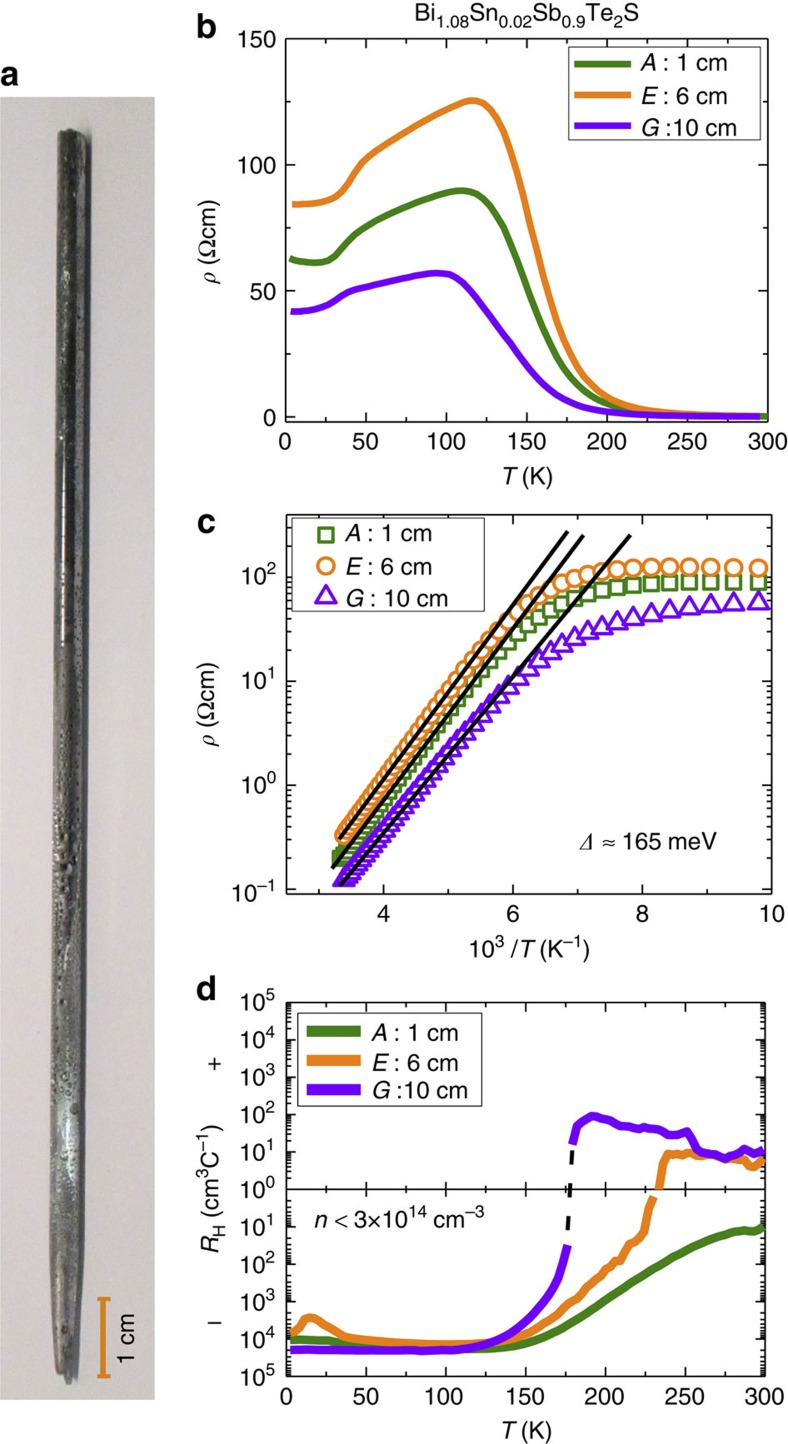
Crystal growth and bulk electronic properties (*ρ* and *n*) of Sn-BSTS. (**a**) The photograph of a crystal boule grown by VBT, an orange line bar represents 1 cm scale. (**b**) The *ρ* versus *T* plots for three freshly cleaved representative samples *A*, *E* and *G*, respectively, taken from the segments at 1, 6 and 10 cm from crystal boule. (**c**) The log *ρ* versus 1/*T* plots for three samples *A*, *E* and *G*, to evaluate thermal activation energy, *Δ*. The straight lines are exponential fits to the high *T* data points. (**d**) The Hall coefficient (*R*_H_) versus *T* plots for the same crystals. The black dotted line is guide to the eye. For samples *E* and *G*, *R*_H_ values show the change of sign from positive to negative.

Our high-quality bulk single crystals of Sn-BSTS were grown by the VBT^24^, a typical crystal boule is shown in Fig. 2a. The perfection of the grown crystals was established by X-ray diffraction and Raman spectroscopy^34^,^35^,^36^ (Supplementary Figs 1 and 2, and Supplementary Notes 1 and 2, respectively). The lattice parameters (Supplementary Table 1), obtained by the refinement of X-ray diffraction pattern of a powder specimen, are in agreement with tetradymite crystal phase. We observed no indication of Sn precipitates in these crystals, even at the nanoscale in our STM studies (described below), consistent with expectations from the very small Sn content used and the successful use of Sn as a resonant level dopant in small percentages in previous studies on tetradymites (see, for example, refs 24, 25, 33). The excellent cleavage characteristics of our single crystals are further indication of the absence of Sn precipitates.

### Bulk transport characterization

The temperature-dependent bulk resistivity (*ρ*) plots (Fig. 2b) of three samples of Sn-BSTS from a 15-cm-long single crystal boule, separated by a total of ∼8 cm along the boule, show that the bulk Sn-BSTS is highly insulating. *ρ* increases exponentially with decreasing *T*, attains maximum values (*∼*125 Ωcm was the largest observed) at ∼100–150 K and starts decreasing with decreasing *T*. This decrease, which has been observed numerous times in previous studies on tetradymite-based materials with low carrier concentrations, is widely taken as an indication that the resistivity of the insulating bulk material has been short-circuited at low temperatures by the metallic TSS^20^,^30^,^37^,^38^. The temperature at which the SSs finally dominate the bulk resistivity depends on intrinsic factors such as the exact defect concentration in the bulk crystal, and the surface and bulk carrier mobilities, and extrinsic factors such as the sample shape. These are the origin of the relatively small differences in behaviour observed here for crystals extracted from different locations in the large, single crystal boule. The point is that the same basic behaviour is seen for crystals extracted from widely varying positions, an indication of the uniformity of the defect chemistry in the large bulk single crystal. The high values of resistivity observed are excellent for a tetradymite; at *T*<100 K, the bulk resistivity must be substantially higher but it cannot be measured directly due to the dominance of the SS conductance. The Log *ρ* versus *T*^−1^ plots (Fig. 2c) exhibit an activated behaviour with the activation energy for transport *Δ*≈165 meV. This *Δ* is very high and is consistent with half of the bulk band gap measured by angle-resolved photoemission spectroscopy (ARPES) (see below) (*Δ*=*E*_g (transport)_/2, thus *E*_g_=330 meV, *E*_g(ARPES)_=350 meV), an indication of the intrinsic nature of the semiconducting Sn-BSTS crystals. The Hall coefficient (*R*_H_) (Fig. 2d) increases with decreasing *T* and attains very high values (∼ −5 × 10^4^ cm^3^ C^−1^) at low *T*. Some samples show a change in sign of *R*_H_ from positive to negative on cooling, which is typical behaviour for highly compensated bulk tetradymites (see, for example, ref. 39), and for *T* <150 K, all samples have negative *R*_H_, indicating *n*-type carrier conduction. At the low *T* transport dominated by SSs, the bulk carrier concentrations must actually be substantially lower than the ∼3 × 10^14^ cm^−3^ that results when one assumes that the conduction is from bulk states only. The feature in *ρ* near *T*∼50 K, where SS transport dominates the conductance, is reproduced in all of the crystals studied and is discussed further below.

### Angle-resolved photoemission spectroscopy

**Figure 3:**
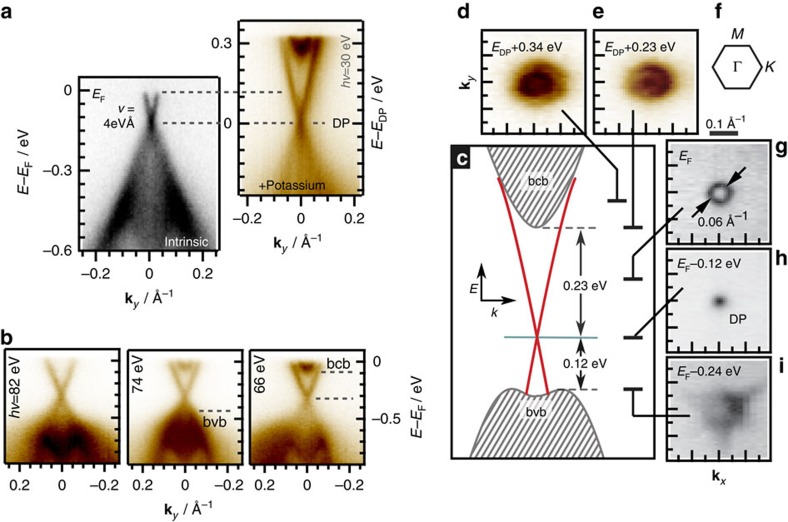
Probing the electronic band structure by ARPES measurements. (**a**) The ARPES-determined band dispersion for **k**_*y*_ along *M*–*Γ*–*M* measured at 30 eV photon energy, at 15 K. The bands were electron doped through deposition of potassium (K), to allow for the determination of the energy of the conduction band minimum. (**b**) Excerpts from the excitation energy dependence (*hv*=82, 74 and 66 eV) study of the photoemission from the electron-doped bands reflect the perpendicular momentum **k**_*z*_ positions of the BVB and BCB extrema. The SSs are unaffected by the change of **k**_*z*_. (**c**) A schematic for the electronic structure of Sn-BSTS in the vicinity of the Fermi energy derived from the ARPES data. Grey dashed lines mark the bulk band gap region, two crossed red lines correspond to the SSs and the turquoise line shows the position of the DP. The constant-energy ARPES maps, referenced relative to the Fermi level *E*_F_ for the pristine sample (**g**–**i**) or the DP *E*_DP_ for the K-deposition-generated electron-doped sample (**d**,**e**), showing a few critical points in the low-energy band structure of Sn-BSTS. (**f**) A schematic for Brillouin zone with high symmetry points.

The low-energy band structure of Sn-BSTS was investigated by ARPES and is shown in Fig. 3. The observations are consistent with what has been seen in other tetradymite-based materials and no indication of the energy levels of the 1% Sn-dopant states is observed. The pristine crystal surface shows conical states in the centre of the surface Brillouin zone that are linearly dispersing (Supplementary Fig. 3 and Supplementary Note 3) from the DP at ∼120 meV below the Fermi level with velocity of 4 eVÅ, forming the tiny circular electron pocket (Fig. 3a). The bands probed by ARPES were then additionally electron doped through the deposition of K atoms on the surface, pushing the chemical potential into states that are not occupied in the pristine crystals. Consistent with previous studies on tetradymites, the conical bands display no photon-energy dependence, a test of the **k**_*z*_ dependence of their energy dispersion, and therefore represent SSs. The photon and **k**-dependent ARPES spectra of the K-deposited crystals enabled the determination of the extrema of the bulk valence and conduction bands that form the energy gap in which the SSs reside, Fig. 3b. The DP was thus found to be isolated from the bulk bands, as the highest BVB states only reach energies of 120 meV below while the lowest energy BCB states appear 230 meV above the DP (these could also be BCB-derived quantum well states of electrons trapped near the surface by band bending; however, as no Rashba splitting is observed, it is likely to be that the band bending is small, and that these states only trace the bottom of the BCB^40^,^41^). These findings are shown schematically in Fig. 3c. The bulk gap is found to be ∼350 meV, consistent with the transport activation energy. *E*_F_ falls in the SSs only and the Fermi surface, shown in Fig. 3g, consists only of a small ring centred at the *Γ* point of the Brillouin zone (Fig. 3f), with the Fermi wave vector **k**_F_=0.03 Å^−1^, for the SS electrons. Constant energy cuts through the band structure (Fig. 3g–i) show that the SSs, as one moves away from the DP, acquire some hexagonal warping and are joined by the six petals of the BVBs some 240 meV below the Fermi level in the pristine sample, or the circular bottom of the BCB when the chemical potential is raised by 110 meV through K deposition (Fig. 3d,e). This is confirmed in our tunnelling microscopy experiments.

### Scanning tunnelling microscopy

**Figure 4:**
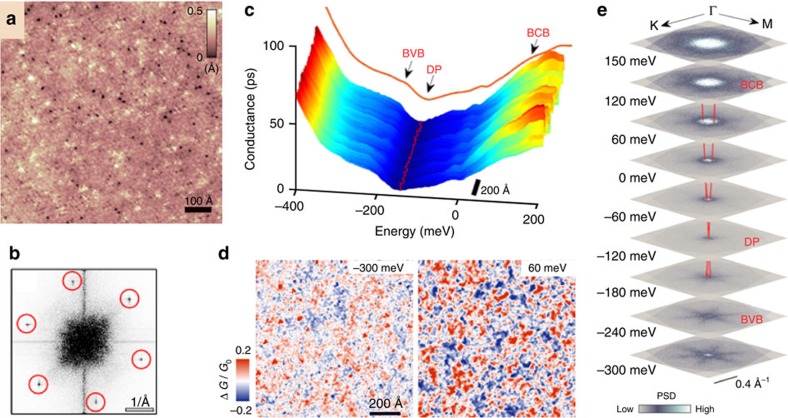
Mapping of electronic conductance and 2D SSs by STM. (**a**) Real space topographic image of the (001) surface of an Sn-BSTS crystal at *V*_bias_=−600 mV and *I*=60 pA tunnelling current. (**b**) Fourier transform of the topographic image revealing the Bragg peaks corresponding to the hexagonal surface structure. (**c**) The differential conductance (*dI*/*dV*) along a line shows only small fluctuations in the electronic surface structure (*V*_bias_=−600 mV and *I*=60 pA). The red line indicates the minima of the spectra, whereas the brown line displays the average spectrum along the line. Arrows point to the approximate positions of the edges of the bulk valence and conduction bands (BVB and BCB), and the position of the DP. (**d**) Real space differential conductance (*dI*/*dV*) maps recorded at −300 and 60 meV with respect to the Fermi level. (**e**) Fourier-transform STS maps at different energies reveal the structure of the quasiparticle scattering on the surface of the crystal. The images were obtained from symmetrized (considering the mirror and rotational symmetry of the underlying crystal) Fourier transforms of *dI*/*dV* conductance maps of size of 1,320 × 1,320 Å (*V*_bias_=−500 mV and *I*=80 pA). The red lines with a slope of 2 eVÅ correspond to the expected dispersion relation that should be seen by STM, based on the ARPES measurements (Fig. 3, *v*_ARPES_=4 eVÅ). The measurements were performed between 30 and 90 K.

The topographic image of the Te-terminated (001) surface of an Sn-BSTS crystal at 30 K displays large atomically flat regions with atomic modulations corresponding to the rhombohedral crystal structure (Fig. 4a); the Fourier transform of this image reflects the hexagonal symmetry of the crystal surface (Fig. 4b). In addition to the Te atoms, several defects are observed^26^. We probed the electronic structure on the surface of Sn-BSTS by using scanning tunnelling spectroscopy (STS)^42^,^43^,^44^,^45^. First, we measured the differential conductance, which is proportional to the local density of states, across the surface along a line of length of 90 nm (Fig. 4c). The spectra show a ‘U' shape curve with a minimum at −120 meV and multiple inflection points. As STS is sensitive to both TSS and bulk bands, to correctly identify the features in the spectra, the STS results are compared with the *E*–**k** structure established by ARPES. We identify the observed changes in the STS spectra at around −200 meV and +120 meV, to be the indication of the edges of the bulk bands. The tunnelling between these energies is dominated by the SSs. The minimum of the conductance corresponds approximately to the DP energy. The DP energy (120 meV below the Fermi energy) is in agreement with the ARPES data measured in different crystals from the same boule, showing the long-range homogeneity of the material. The variation in the position of the minimum energy along the scan line is small (with 2.4 meV s.d.), which suggests that the chemical potential is homogeneous at the nanometer length scale and the DP energy is not affected significantly by the defects on the surface. However, as the energy is tuned away from the DP energy, the density of states develops significant modulations. These inhomogeneities in the spectra are mainly the result of the disordered Sb–Bi distribution in the layers, which leads to enhanced quasi-particle interference on the surface. Scattering of the surface electronic states was examined by recording differential conductance maps at various energies (Fig. 4d). Although the conductance maps are strongly influenced by the Sb-Bi disorder, the Fourier transforms of the maps capture the signal corresponding to the scattering of TSS and reveal a linearly dispersing scattering cone between the BCB and BVB (Fig. 4e). To compare the STM and ARPES data, we note that scattering between the opposite sides of the Dirac cone is prohibited by time-reversal symmetry. In the case of a single Dirac cone, the prohibition suppresses the amplitude of the largest scattering wave vectors backscattering, resulting in a scattering cone with a smaller diameter. However, unambiguously resolving the diameter change is beyond our measurement resolution. Thus, we simply approximate the observed scattering cone as a manifold of vectors connecting regions of large density of states measured by ARPES. In this picture, the boundary of the observed energy-momentum structure for a single linear dispersing band has half of the slope as measured by ARPES. The expected energy–momentum relation is indicated as a red line (Fig. 4e), in good agreement with our ARPES measurement.

### Magnetoresistance and 2D SSs

**Figure 5:**
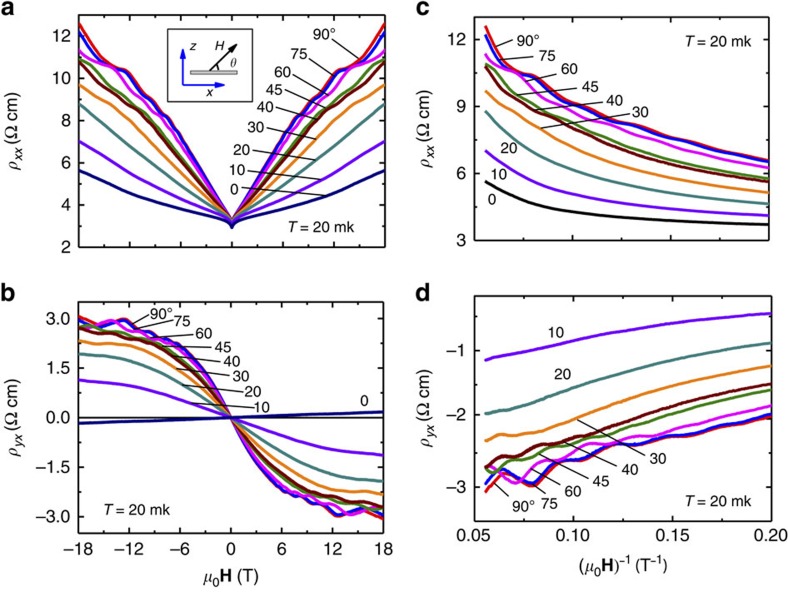
MR and Hall transport characterization of Sn-BSTS. (**a**) The MR and (**b**) Hall plots, and their dependence on the angle (*θ*) between **H** and the current in the sample; all the measurements were performed at 20 mK. A schematic in (**a**) shows the direction of **H** in *x*–*z* plane with respect to current in sample plane along *x*. (**c**,**d**), *ρ*_*xx*_(**H**, *θ*) and *ρ*_*yx*_(**H**, *θ*) versus (*μ*_0_**H**)^−1^ plots as a function of *θ*.

**Figure 6:**
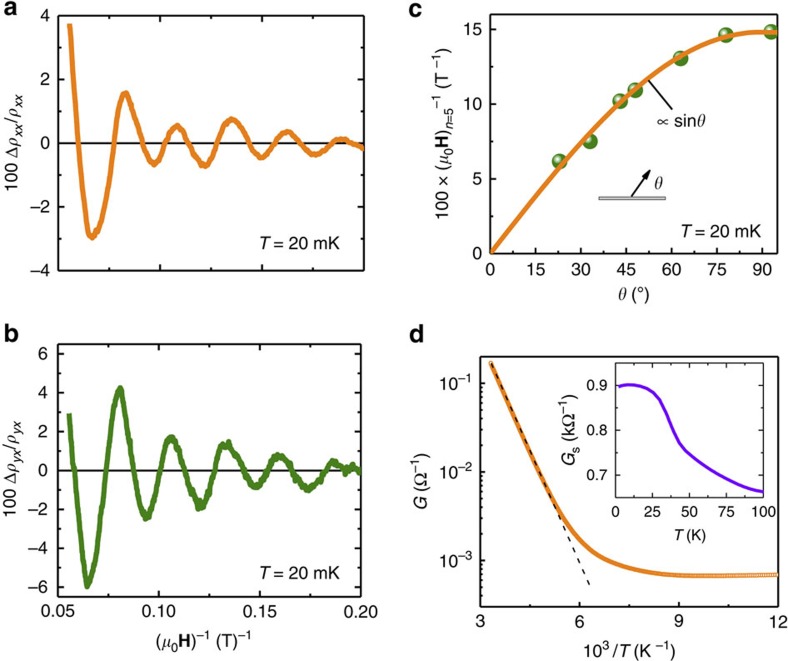
The Shubnikov–de Hass (SdH) oscillations and 2D SSs. (**a**,**b**) SdH oscillations extracted from *ρ*_*xx*_ and *ρ*_*yx*_ for *θ*=90°, respectively. (**c**) The *θ* dependence of (*μ*_0_**H**)^−1^_*n*=5_ peak of SdH oscillations for *ρ*_*xx*_. (**d**) Temperature dependence of the conductivity, for the most insulating crystal of Sn-BSTS studied, plotted as a function of inverse temperature. Inset: The *G*_s_ versus *T* plot for *T*<100 K, extracted from *G*.

To study the availability of the TSS for charge transport experiments and potential applications of Sn-BSTS in advanced devices, we performed transport measurements on crystals at 20 mK, under magnetic fields up to 18 T. Figure 5a,b are the magnetoresistance (MR) (*ρ*_*xx*_ versus **H**) and Hall (*ρ*_*yx*_ versus **H**) plots, respectively, measured by tilting **H** in the *x–z* plane (*z* is the direction of the surface normal and *x* is the direction of current flow in the basal plane). The *ρ*_*yx*_ versus **H** plots show that the charge transport is mainly due to electrons. Both *ρ*_*xx*_ and *ρ*_*yx*_ exhibit strong quantum oscillations at *θ*=90°, which gradually die out as *θ* approaches 0°. To visualize the oscillations and study their origin, the angular dependence of *ρ*_*xx*_ and *ρ*_*yx*_ are plotted as a function of **H**^−1^ (Fig. 5c,d). The Shubnikov-de Haas oscillations are extracted from *ρ*_xx_ and *ρ*_yx_, and the resulting difference resistivity (*Δρ*) plots are shown in Fig. 6a,b; the oscillations are as large as ∼6%, with a low frequency. For a particular oscillation peak (*n*=5) the field value corresponding to the peak position ((*μ*_0_**H**)^−1^_n=5_) is plotted with tilt angle (Fig. 6c). It is clear that the peak shift is proportional to sin*θ*, which has been widely taken as a reflection of the 2D TSS origin of the oscillations (see, for example, refs 16, 18). The Fermi surface cross-section area (*S*_F_) is calculated as 37 T. The 2D SS carrier density *n*_s_ is 8.93 × 10^11^ cm^−2^ and the SS carriers are *n*-type. Using the ARPES Fermi velocity (*v*_F_=4eVÅ), the Fermi surface in this crystal is found to be 134 meV above the DP, consistent with ARPES and STM measurements, again an indication of the homogeneity of the material over macroscopic distances in the single crystal. (For more clarity concerning the 2D nature of the states, *ρ*_*xx*_ and *ρ*_*yx*_ are plotted as a function of (*μ*_0_**H**sin*θ*)^−1^ in Supplementary Fig. 4; the consistent shift of the peak positions of oscillations confirms that these oscillations are owing to 2D conduction (Supplementary Note 4).) We note that quantum oscillations due to the SSs are seen for the vast majority of samples that we have cut from the crystal boules of Sn-BSTS. Although to the authors' knowledge there has been no systematic study of when such oscillations are present in tetradymites and when they are absent, their presence must be related to the crystal surface quality, otherwise they would be observed more frequently.

The TI crystals can be considered as having parallel conductance paths, with the measured electrical conductance being a sum of bulk (*G*_bulk_) and surface (*G*_s_) conductances, *G*=*G*_bulk_+*G*_s_ (ref. 39). With this in mind, the Sn-BSTS crystals allow for the SS conductance to be characterized over a wide temperature range. Taking the data for a very high bulk resistivity crystal, *G* is plotted versus *T*^−1^ in Fig. 6d. At high *T*, *G*_bulk_ is dominant; it decreases exponentially as *T* decreases and for a wide range of temperatures, in the range of 150–100 K, both surface and bulk carriers are contributing significantly to the observed transport. For temperature below 100 K, surface carriers probably dominate the transport. *G*_s_ can then be estimated at low temperature, where the influence of the bulk is less significant. The estimated *T* dependence of *G*_s_ in the range 4–100 K is plotted in the inset of Fig. 6d. *G*_s_ shows an unusual step at ∼50 K. This feature may either be an experimental artefact or purely due to TSS behaviour, or due to a structural phase transition in the underlying bulk crystal. Synchrotron diffraction analysis at low *T* (Supplementary Fig. 5 and Supplementary Note 5) rules out the possibility of a bulk structural phase transition and the STM images taken at low *T* do not show any sign of a surface reconstruction. Therefore, this anomaly cannot reflect the presence of structural transitions either on the surface or in the bulk material. We speculate that it may reflect the sensitivity of conductance of TSS on Sn-BSTS to the adsorption of a residual active gas (for example, H_2_ or O_2_) in the experimental chamber.

## Discussion

We have shown that highly bulk insulating TI crystals of Bi_1.08_Sn_0.02_Sb_0.9_Te_2_S, with intrinsic semiconductor characteristics (*Δ* ∼165 meV and bulk carrier density *n*<3 × 10^−14^ cm^−3^ at low temperatures) can be successfully grown by the VBT, with nearly uniform properties over 8 cm or more of a large single crystal boule. The bulk band gap is consistently estimated to be ∼350 meV by spectroscopy and transport methods, and the DP is found to be completely exposed in the gap, ∼120 meV above the valence band maximum and ∼230 meV below the conduction band minimum. The chemical potential for these crystals consistently falls in the SSs only, at an energy of ∼120 meV above the DP. Quantum oscillations from the TSS electrons are reproducibly observed. The SS carriers in Sn-BSTS are *n*-type, which are preferable for charge transport experiments and potential spintronics applications. Our results lead us to conclude that Sn-BSTS is an excellent tetradymite-type bulk single crystal TI, and that it will be highly advantageous for use in future experimental studies of TSSs, especially for the characterization of SS quantum transport very close to the DP.

## Methods

### Crystal growth

The bulk single crystals were grown by the VBT. The required amounts (according to the formula: Bi_1.08_Sn_0.02_Sb_0.9_Te_2_S) of elements with high purities (5 N) were placed in carbon-coated, bottom-pointed quartz ampoules with an inner diameter of 4 mm. The as-received high-purity starting materials (Bi, Sb and Te) were further purified by heating under vacuum of∼10^−5^ mbar at 950 °C in the presence of carbon, in sealed quartz tubes. The crystal growth ampoules were sealed under a vacuum of∼10^−5^ mbar. The crystal growth was performed at ∼700 °C with a translation rate of ampoule of 1 mm h^−1^ through the crystallization zone^24^ and ∼15-cm-long crystal boules were obtained.

### Crystalline quality

The crystals extracted from the single crystal boule were studied for phase identification on a laboratory X-ray diffractometer (Bruker Eco D8 Advance) equipped with CuK*α* source and LYNXEYE XE detector. To check the homogeneity of the crystal structure we also performed Raman microscopy at ambient temperature using 10 μW from a 532-nm excitation laser, focused to 1 μm spot. Temperature-dependent synchrotron powder X-ray diffraction measurements were performed on beamline 11-BM at the Advanced Photon Source, to probe the bulk crystal structure at low temperature.

### Bulk resistivity and carrier density

The bulk resistivity and carrier density were measured respectively in four-probe and Hall configurations on a Quantum Design Physical Properties Measurement System. Samples were in the rectangular shape with thickness of ∼0.5 mm. The electrical contacts were made on the (001) surface through Pt wires with silver paint. The Hall data were recorded by applying magnetic field of 1 T normal to the (001) sample surface and the current flow was in the basal plane.

### ARPES and STM band structure analysis

The electronic band structure was established by ARPES using a Scienta SES-3000 electron spectrometer set to the resolution of 12 meV and 0.1° at beamline 12.0.1 of the Advanced Light Source. Photon energies were in the range of 30–100 eV. Samples were cleaved at 15 K in ultrahigh vacuum of 5 × 10^−9^ Pa and all the data were collected at 15 K. The STM measurements were performed on the (001) crystal surface in the temperature range of 30–90 K, on a home-built variable temperature STM.

### MR and Hall effect

Angular-dependent MR and Hall data were obtained in a dilution refrigerator at *T*=20 mK at applied fields up to *μ*_0_**H**=18 T, using standard six contact measurements. The Shubnikov-de Haas quantum oscillations were obtained by subtracting the background from the data. The thickness of samples used for measurements was ∼50 μm.

## Additional information

**How to cite this article:** Kushwaha, S. K. *et al*. Sn-doped Bi_1.1_Sb_0.9_Te_2_S bulk crystal topological insulator with excellent properties. *Nat. Commun.* 7:11456 doi: 10.1038/ncomms11456 (2016).

## Supplementary Material

Supplementary Information Supplementary Figures 1-5, Supplementary Table 1, Supplementary Notes 1-5 and Supplementary References
