# Supplementary material for: Sn-doped Bi1.1Sb0.9Te2S bulk crystal topological insulator with excellent properties
Source: Nat Commun. 2016 Apr 27;7:11456. doi: 10.1038/ncomms11456 (PMC4853473; doi:10.1038/ncomms11456)
Supplement: Supplementary Information — Supplementary Figures 1-5, Supplementary Table 1, Supplementary Notes 1-5 and Supplementary References [file ncomms11456-s1.pdf]

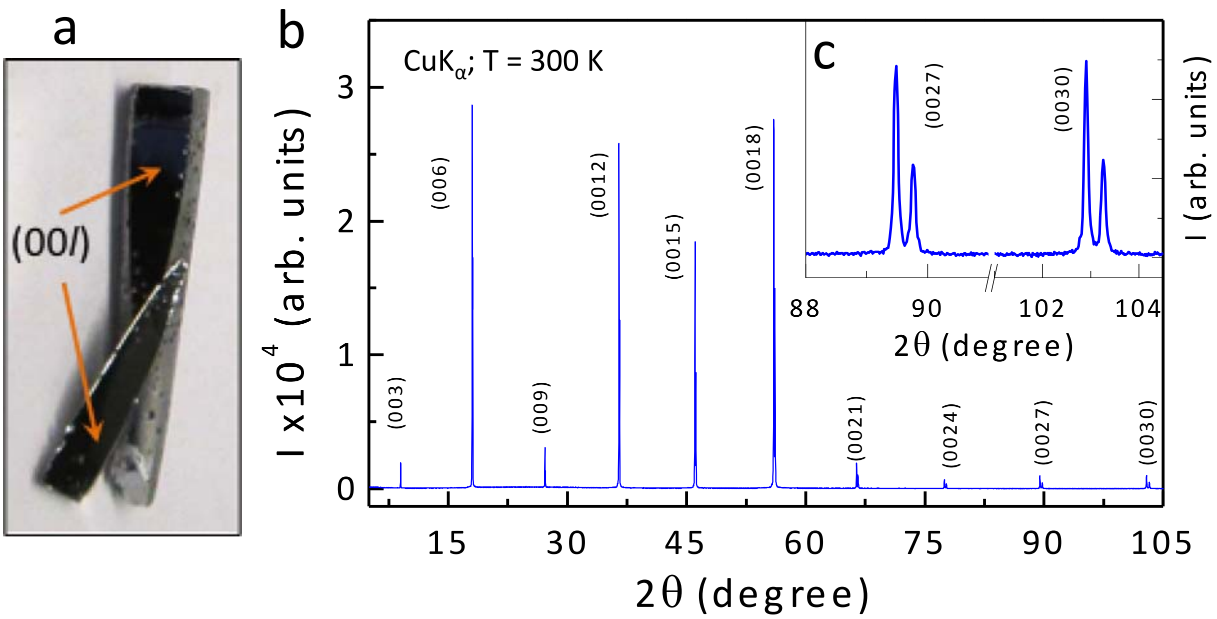

Supplementary Figure 1 | Crystal with (00*l*) cleavage along plane and X-ray diffraction

(a) The photograph of crystal specimen cleaved along (00*l*) crystallographic planes. **(b)** The X-ray diffractogram recorded in Bragg-Brentano geometry for a (001) crystal face, exhibiting the diffraction peaks corresponding only to the (00*l*) planes. **(c)** The excellent $K\alpha_1$-α_2 splitting of the higher order diffraction peaks is observed, testifying to the high crystalline quality. A break in applied on the x-axis for an angular range.

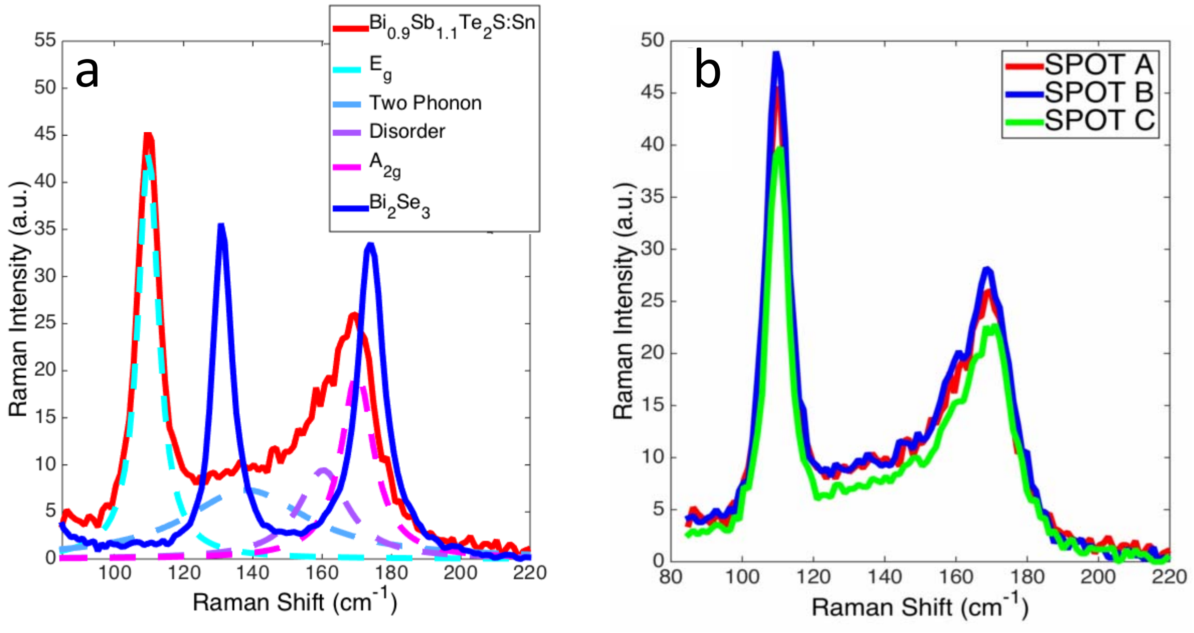

Supplementary Figure 2 | The Raman spectra for E_g and A_{2g} phonons.

(a) Raman spectra measured on the (001) surface of Sn-BSTS and Bi_2Se_3 showing comparable mode widths. This is further confirmed by Lorentzian fits to the spectra, which include a defect induced mode and a two-phonon excitation. **(b)** Raman spectra are taken at 3 different locations named as SPOT A, SPOT B and SPOT C, 2mm apart, showing the excellent uniformity of the crystal, through reproducibility of the phonon spectra.

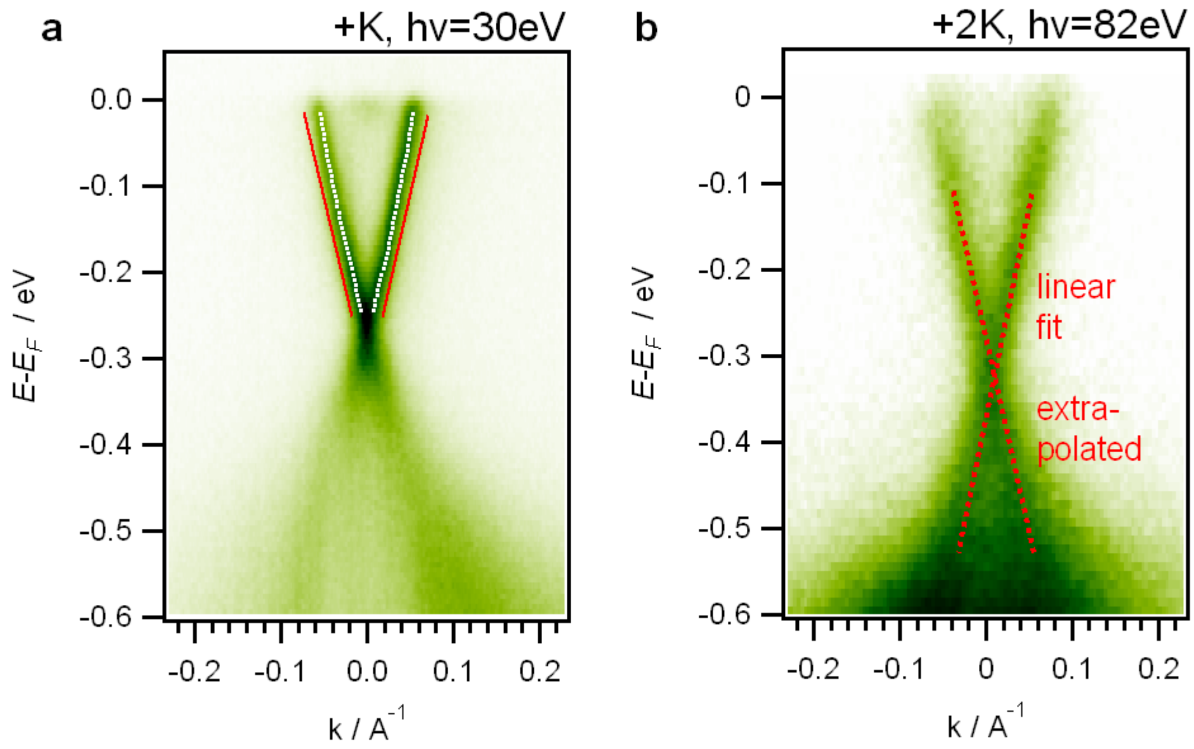

Supplementary Figure 3 | The ARPES spectra near Dirac cone

(a) Shows an extraction of the upper Dirac cone dispersions by using the *k*-positions (shown white dots) of the maxima in intensity of the ARPES spectrum for the potassium-deposited electron-doped sample. The red lines show the fitting of dispersions. **(b)** An elucidation of lower Dirac bands, by extrapolation of linear bands.

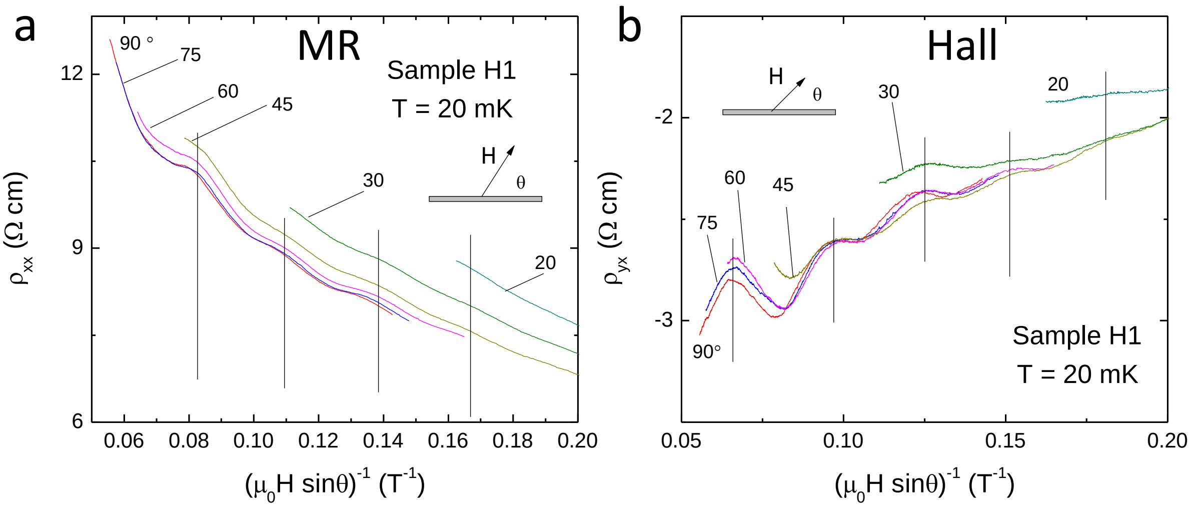

Supplementary Figure 4 | The ρ_{xx} and ρ_{yx} vs. $(\mu_0 H\sin\theta)^{-1}$ plots

(a) and **(b)** are the magnetoresistance (MR) and Hall plots with inverse of magnetic field for one of the samples characterized. The schematic in the inset of figures shows the angle, θ, between magnetic field (represented by an arrow) and current in sample plane.

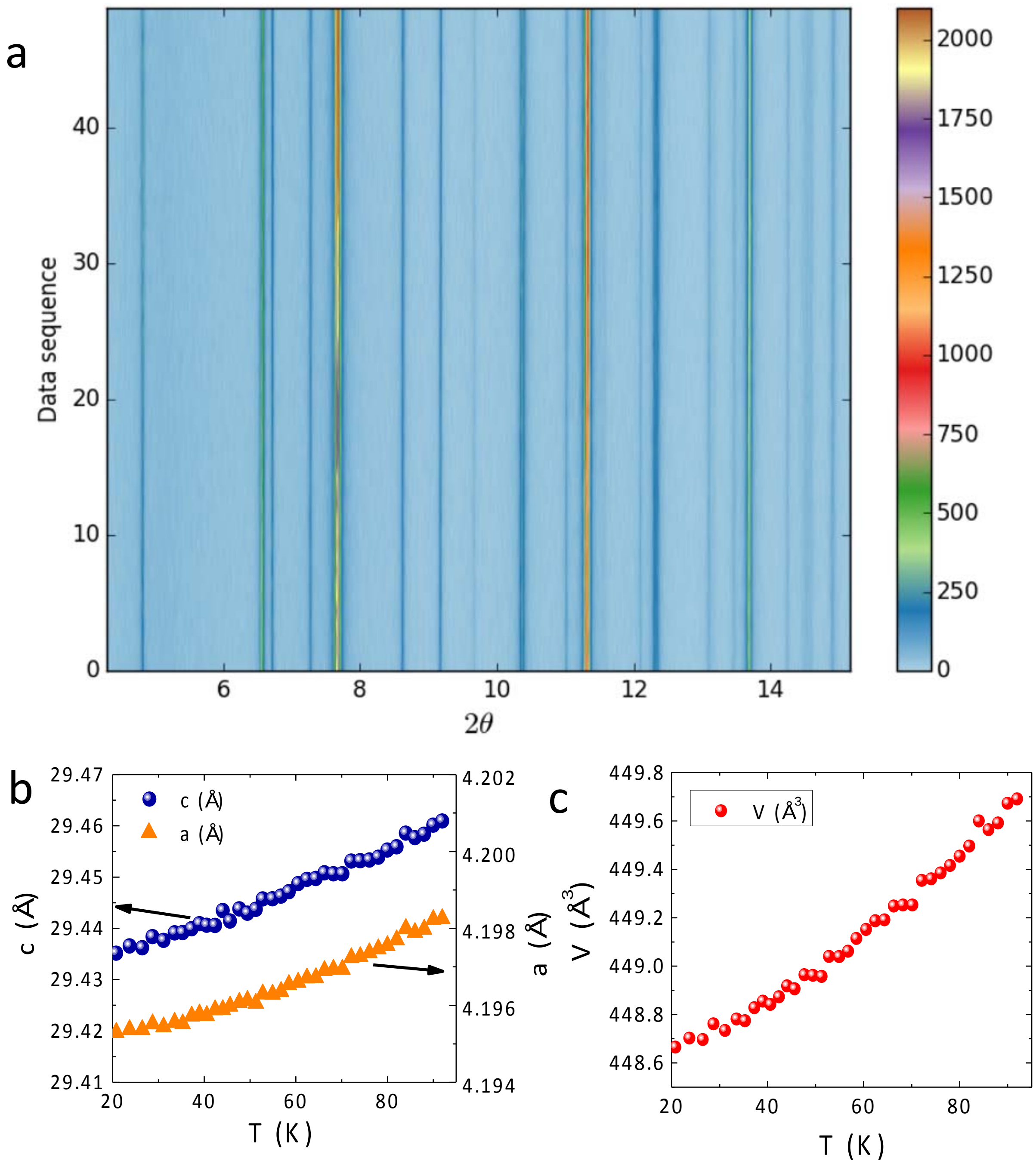

Supplementary Figure 5 | The synchrotron powder X-ray diffraction and lattice parameters

(a) The temperature dependent evolution of the synchrotron powder X-ray diffraction pattern for a powdered specimen of Sn-BSTS (λ = 0.413727 Å) plotted for the data sequence recorded in the

temperature range of 2 – 90 K: data sequence-1 (vertical axis) is recorded at the temperature 2 K and data sequence-50 is recorded at 90 K. **(b)** The lattice parameters (*a* and *c*) determined by least squares fits to the observed diffraction patterns. **(c)** Cell volume plots for T = 20-90 K. No anomalous behavior is observed at ~50 K for any of the bulk crystal parameters. (error bars are smaller than the plotted points) The results indicate that no bulk structural transition occurs in Sn-BSTS in this temperature range.

Supplementary Table 1

a = 4.21053(5) Å			c = 29.55453(25) Å		
Atom	Wyck.	*x*	*y*	*z*	*Occ.*
Bi	6*c*	0	0	0.39173(1)	0.57(1)
Sb	6*c*	0	0	0.39173(1)	0.43(1)
Te	6*c*	0	0	0.21509(2)	1
S	3*a*	0	0	0	1
Rp = 10.2	*Rwp* = 13.1		*Rexp* = 6.63	χ^2 = 3.89	

The refined lattice parameters and atomic coordinates

Supplementary Note 1 | Bulk crystal growth and structure analysis

The high quality single crystal boule (~15 cm long) of $Bi_{1.08}Sn_{0.02}Sb_{0.9}Te_2S$ (Sn-BSTS) (Figure 2**a** of main manuscript) was grown by the VBT growth method[1]. The crystal boule was cut into sections of ~1.5 cm length and named in alphabetic order (A, B, C, … , H), where A represents the first-to-crystallize section in the pointed region of the ampoule, Supplementary Figure 1**a** shows single crystal specimen with cleaving along the (00*l*) plane, with nice flat and shiny surfaces. Supplementary Figure 1**b** displays a diffractogram, recorded in flat-plate reflection geometry for a single crystal specimen (Supplementary Figure 1**a**). The pattern contains the

diffraction peaks corresponding to only (00*l*) planes of very high index. The peaks are very sharp and indicate the high quality of the crystal, which is further evidenced in the very clean $K\alpha_1$ - $K\alpha_2$ splitting of the higher order diffraction peaks (Supplementary Figure 1**c**). This feature reflects the perfection of ordering of S and Te layers and the uniformity of the Sb-Bi distribution in the crystal – a non-uniform composition would broaden the peaks and the α_1- α_2 splitting would be degraded. The crystal structure was analyzed by powder X-ray diffraction on a crushed crystal, the diffraction pattern was modeled and refined using Rietveld method based on a rhombohedral cell derived from $Bi_2Te_{1.6}S_{1.4}$.[2] The refined unit cell information, including cell parameters, atomic positions and occupancies are listed in Supplementary Table 1. The unit cell dimensions, a = 4.21053(5) Å and c = 29.5545(3) Å, are in good agreement with earlier reported tetradymite phases[3]. These lattice constants are slightly different from those found for the composition $Bi_2Te_{1.6}S_{1.4}$, which has partial replacement of Te atoms in the outer layers of the quintuple layer by S atoms that leads to disorders in the quintuple layers[2]. The atomic level lattice defects of freshly cleaved (001) surface were analyzed by recording STM topographic images (Figure 4**a** of main manuscript), which displayed large atomically flat regions with atomic modulations corresponding to the rhombohedral crystal structure. As expected for this type of crystal structure, the cleaving results in Te-terminated (001) surfaces. In addition to the Te atoms, the surface showed several defects[4].

Supplementary Note 2 | Raman spectroscopy analysis

The freshly cleaved (001) surface of a crystal of Sn-BSTS was subjected to analysis of its long-range lattice perfection by Raman spectroscopy. The recorded Raman spectra (Supplementary Figure 2**a** & **b**) are fit with four Lorentzians. Two of these modes are the E_g and A_{2g} phonons

typically found in this range for $(Bi_{1-y}Sb_y)_2Te_{3-x}Se_x$,[5,6] with the higher energy mode splitting as is typically seen for occupancy of the three X sites in the M_2X_3 quintuple layer by different ions (i.e. Te and Se or Te and S). The fourth, very broad mode is also common in these materials, and results from two-phonon scattering. The widths of the E_g and A_{2g} modes in Sn-BSTS (~8 cm^{-1} & 11 cm^{-1} respectively) are comparable with those seen in the chemically simpler material Bi_2Se_3 (~7 cm^{-1} & 9 cm^{-1}), indicating the excellent quality of the Sn-BSTS crystal. Furthermore, the phonon positions and widths are highly reproducible from place to place in the crystal, as seen in the Supplementary Figure 2**b**, where we show spectra measured from three locations 2 mm apart.

Supplementary Note 3 | Linear dispersion of surface states in the vicinity of Dirac point

We comment on the linear dispersion of the E vs. **k** relations for the Topological Surface States in the vicinity of the Dirac point in Sn-BSTS. This is illustrated in Supplementary Figure 3. We have extracted the upper cone dispersion by using the **k**-positions (white dots in Supplementary Figure 3**a**) of the maximum intensity of the ARPES spectra for the potassium-deposited electron doped sample and fitted them to a line (red, slightly offset in **k**, in Supplementary Figure 3**a**, to reduce clutter). We then extrapolated the linear bands, and overlaid them onto an ARPES spectrum recorded at an energy that makes the lower bands visible as well. One can see from the Supplementary Figure 3**b** that, within a few tenths of an eV of the Dirac point and within our experimental precision, the upper and lower bands follow the same linear dispersion very well.

Supplementary Note 4 | The angular dependence of quantum oscillations

To confirm that the quantum oscillations observed in MR and Hall measurements (Figure 5**a** and 5**b**, of the main manuscript) are only from the 2-dimensional surface states, the ρ_{xx} and ρ_{yx} oscillations *vs.* inverse magnetic field were plotted for different θ, the angle between applied magnetic field and current in sample (Supplementary Figures 4**a** and **b**). Both kinds of plots show a nice peak shift with θ. The amplitude of oscillations gradually increased as θ approaches to 90 degrees. The angular dependent shift in the peak positions of oscillations confirms that the oscillations are only due to the 2D surface states.

Supplementary Note 5 | Low temperature synchrotron X-ray diffraction analysis

An anomalous feature is seen near 50 K in the temperature dependence of the surface conductance (Figure 6**e** of main manuscript text), which may either be a feature purely due to the surface state behavior, or a reflection of the presence of a structural phase transition in the underlying bulk crystal. To investigate this latter possibility, we investigated the crystal structure of Sn-BSTS in the temperature range of 2-90 K by high-resolution synchrotron powder X-ray diffraction. A plot showing the general temperature dependence of the observed diffraction patterns is shown Supplementary Figure 5**a**. No structural transitions are observed in this temperature range– neither in the crystal symmetry nor in the appearance of extra diffraction peaks. According to the very high resolution and high dynamic range of the synchrotron diffraction experiments, Sn-BSTS remains rhombohedral and in the tetradymite structure type to low temperatures. Further, the bulk lattice parameters (*a* and *c*) of the rhombohedral cell can be followed at high precision as a function of *T* from 20 K to 90 K (Supplementary Figure 5**b** and **c**). They do not exhibit any anomaly at $T \sim 50$ K. From this data it is clear that the observed

anomalies in the transport data cannot be attributed to anomalies in the structure of the underlying bulk phase. Further, the STM and APRES measurements at temperatures below 50 K did not reveal the presence of structural anomalies on the basal plane crystal surface.

Supplementary References

1. Kushwaha, S. K., et al. Comparison of Sn-doped and nonstoichiometric vertical-Bridgman-grown crystals of the topological insulator Bi_2Te_2Se. *J. Appl. Phys.* **115**, 143708-143713 (2014).

2. Ji, H., et al. $Bi_2Te_{1.6}S_{1.4}$: A topological insulator in the tetradymite family. *Phys. Rev. B* **85**, 201103(R) (2012).

3. Neumann, G., Seideġġer, R. Über die Existenz verschiedener Phasen im quasibinären Szstem Bi_2Se_2 - Bi_2S_3. *Helvetica Physica Acta* **40**, 293 (1967).

4. Jia, S., et al. Defects and high bulk resistivities in the Bi-rich tetradymite topological insulator $Bi_{2+x}Te_{2-x}Se$. *Phys. Rev. B* **86**, 165119 (2012).

5. Richter, W., Becker, C. R. A Raman and far-infrared investigation of phonons in the rhombohedral V_2–VI_3 compounds Bi_2Te_3, Bi_2Se_3, Sb_2Te_3 and $Bi_2(Te_{1-x}Se_x)_3$ ($0 < x < 1$), $(Bi_{1-y}Sb_y)_2Te_3$ ($0 < y < 1$). *Physica Status Solidi (B)* **84**, 619 (1977).

6. Zhao, S. Y. F., et al. Fabrication and Characterization of Topological Insulator Bi_2Se_3 nanocrystals. *Appl. Phys. Lett.* **98**, 141911 (2011).
